# Benefits and Risks of Pesticide Testing on Humans

**DOI:** 10.1289/ehp.113-1314936

**Published:** 2005-12

**Authors:** Herbert L. Needleman, J. Routt Reigart, Philip Landrigan, Jennifer Sass, Cynthia Bearer

**Affiliations:** School of Medicine, University of Pittsburgh, Pittsburgh, Pennsylvania, E-mail: hlnlead@pitt.edu; Medical University of South Carolina, Charleston, South Carolina; Department of Community Medicine, Mt. Sinai School of Medicine, New York, New York; Natural Resources Defense Council, Washington, DC; School of Medicine, Case Western Reserve University, Cleveland, Ohio

In their review of the history of the U.S. Environmental Protection Agency’s (EPA) response to the question of human testing of pesticides, Resnick and Portier (2005) argued that the benefits of such testing outweigh the hazards, and they attempted to refute claims that human testing is both unproductive and unethical. We consider their arguments vague, tendentious, and essentially incorrect.

Unanimously passed by both houses of Congress in 1996, with the support of pesticide manufacturers, pediatricians, and the environmental community, the Food Quality Protection Act (FQPA 1996) added a 10-fold child protective safety factor in choosing a reference dose to two earlier factors, one employed to accommodate the difference between animals and humans and one to accommodate the variance among adults. The single stimulus behind the FQPA was the growing evidence of increased childhood vulnerability, and the single reason for its unanimous, bipartisan passage was to protect children.

The pesticide industry quickly mounted a two-pronged attack on the U.S. EPA’s new guidelines and safety factors (U.S. EPA 2000), arguing that children were not more sensitive than adults. At the same time, they launched studies in which organophosphate pesticides were administered to adult “ volunteers.” This was a palpable effort to circumvent and weaken the 10-fold human/animal safety factor, and it flouted the intent of the law to stimulate the generation of data on the developmental and pediatric toxicity of pesticides. Resnik and Portier (2005), in a curious shift of responsibility, indict the FQPA as a factor in stimulating human studies with their claim that

A law that was intended to provide additional safety protection for children had the unintended effect of encouraging some companies to test toxic compounds on human beings to avoid the regulatory impact of the law.

With few exceptions the U.S. EPA has failed to use the mandated 10X factor. In June 2002 the U.S. EPA issued its cumulative assessment of the organophosphate pesticides (OPs), determining that for the 30 OPs reviewed, a 1X safety factor (that is, no factor) was used for three OPs and one metabolite, and a 3X reduction was used for the others (U.S. EPA 2002). At no time was a 10X factor used, despite the fact that the U.S. EPA possessed developmental neurotoxicity data for only 6 of the 30 OPs at the time of its assessment

In 1998 the U.S. EPA convened a special committee consisting of members of the Science Advisory Board, the Science Advisory Panel for pesticides, and outside ethicists to examine the ethics of human testing. The committee had two meetings separated by 12 months, and after five drafts, adopted a report that accepted human testing subject to rigorous or severe limitations (U.S. EPA 2000). The two pediatricians on the committee (H.L.N. and R.R.) filed a minority report that became part of the record because we objected to procedural and scientific solecisms. Resnik and Portier (2005) did not mention this report, even though Portier was a member of that committee.

Resnik and Portier (2005) recognize that past industry studies are scientifically unacceptable. They directed their comments to future, yet-to-be-specified studies. Such studies, they stated, “can be conducted only if they meet strict scientific and ethical standards and provide public health or environmental benefits.” Resnik and Portier (2005) also stated that studies of adult volunteers “could yield knowledge about the toxic effects on humans, which could promote human health” (National Research Council 2004). The reader is left to wonder what toxic effects would be better understood through human studies, or what health benefits could accrue from short-term volunteer studies. Resnik and Portier (2005) mentioned neither children’s health nor developmental toxicity. Instead they proffered the dubious hope that studies could result in stricter safety standards or new legislation that could result in reduced pesticide exposure. In today’s regulatory climate, this must be considered a slim possibility. Once more the future standards and laws remain unspecified.

Two major issues in human testing are the relevance of data obtained from adult exposure to risk estimates for children, and the scientific validity of short-term human studies as predictors of health outcomes such as neurodevelopmental deficits and carcinogenesis. It is axiomatic that a study that is poorly designed and cannot produce valid conclusions is unethical on this ground alone.

The provenance of the FQPA (1996) emerged from the growing realization that children are vastly different from adults and that the developing organism, while it is laying down and pruning back neural connections, is much more sensitive to neurotoxicants than fully formed organisms. What could possibly be learned about the risk to this group from studying the effects of toxicants on adults? Resnik and Portier (2005) did not attempt to address this question, but the answer is, very little.

One of the critical issues in evaluating the scientific validity of a study design is statistical power. On this basis alone human studies have failed. A study with inadequate power to find an effect is by definition unethical. This type of study submits subjects to some risk while providing no scientific information. There are roughly 19 million children in the United States ≤5 years of age. If a toxicant harmed 1 child in 1,000, that would place 19,000 children at risk nationwide. A study with adequate power to detect an increase in deficit from 1% to 2% would require 3,017 subjects in each group to yield a power of 0.8, at α = 0.05. Past industry studies with sample sizes < 50 had about a 3% chance of finding an effect if it were present. No mention of this power finding was made by Resnik and Portier (2005), although this was published by the U.S. EPA Science Advisory Board/Federal Insecticide, Fungicide, and Rodenticide Act (FIFRA) Science Advisory Panel (U.S. EPA 2000) on which Portier served as a contributing member.

Resnik and Portier’s article (Resnik and Portier 2005), when examined in light of specificity, completeness, and relevance to the health of children, fails on all points. It asks the reader to accept unspecified studies on adults as productive of unspecified benefits to human health. The principal toxic target, the health of children, remains unspoken and out of awareness.
